# An alternating renewal process describes the buildup of perceptual segregation

**DOI:** 10.3389/fncom.2014.00166

**Published:** 2015-01-07

**Authors:** Sara A. Steele, Daniel Tranchina, John Rinzel

**Affiliations:** ^1^Center for Neural Science, New York UniversityNew York, NY, USA; ^2^Courant Institute for Mathematical Sciences, New York UniversityNew York, NY, USA; ^3^Department of Biology, New York UniversityNew York, NY, USA

**Keywords:** stream segregation, alternating renewal process, bistable perception, perceptual dynamics, perceptual organization, buildup

## Abstract

For some ambiguous scenes perceptual conflict arises between integration and segregation. Initially, all stimulus features seem integrated. Then abruptly, perhaps after a few seconds, a segregated percept emerges. For example, segregation of acoustic features into streams may require several seconds. In behavioral experiments, when a subject's reports of stream segregation are averaged over repeated trials, one obtains a buildup function, a smooth time course for segregation probability. The buildup function has been said to reflect an underlying mechanism of evidence accumulation or adaptation. During long duration stimuli perception may alternate between integration and segregation. We present a statistical model based on an alternating renewal process (ARP) that generates buildup functions without an accumulative process. In our model, perception alternates during a trial between different groupings, as in perceptual bistability, with random and independent dominance durations sampled from different percept-specific probability distributions. Using this theory, we describe the short-term dynamics of buildup observed on short trials in terms of the long-term statistics of percept durations for the two alternating perceptual organizations. Our statistical-dynamics model describes well the buildup functions and alternations in simulations of pseudo-mechanistic neuronal network models with percept-selective populations competing through mutual inhibition. Even though the competition model can show history dependence through slow adaptation, our statistical switching model, that neglects history, predicts well the buildup function. We propose that accumulation is not a necessary feature to produce buildup. Generally, if alternations between two states exhibit independent durations with stationary statistics then the associated buildup function can be described by the statistical dynamics of an ARP.

## Introduction

**Figure 1:**
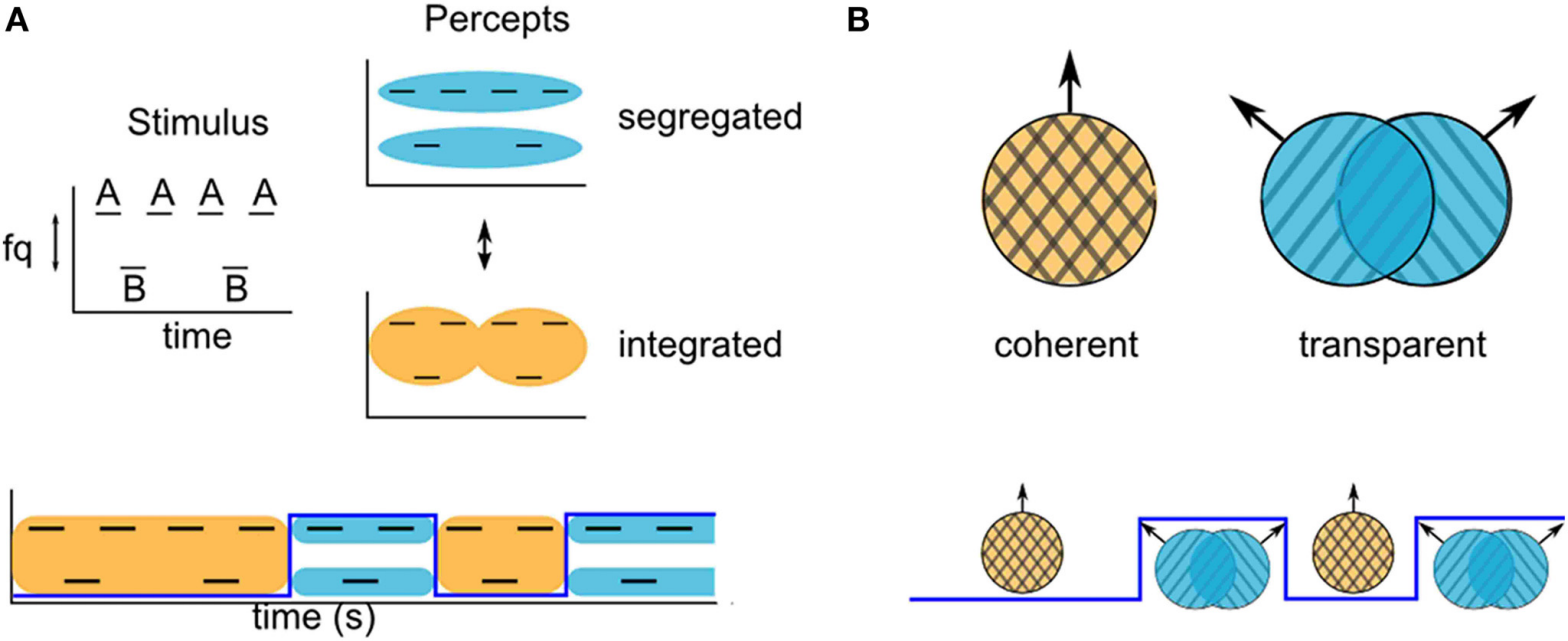
**Examples of stimuli that can produce ambiguous grouping. (A)** Van Noorden triplets with ambiguous stream segregation. Listeners report alternations between hearing integration (bottom, orange) and segregation (top, blue) of the component tone frequencies. **(B)** Moving gratings at certain angles can produce ambiguous motion. Observers report alternations between coherent and transparent motion of the component gratings.

For some stimuli in the auditory and visual modalities with ambiguous grouping cues, subjects report experiencing alternations between grouped and split perceptual organizations, with an initial grouped percept. For example, a widely used paradigm in auditory stream segregation uses the two-tone triplet stimulus, ABA-ABA-… (Van Noorden, 1975), where A and B refer to tones at different frequencies, and-represents a silent interval. Depending on the relative tone-frequency and presentation rate, listeners to a presentation of fixed length may be more likely to report perceiving either triplet patterns grouped into a galloping rhythm, or two segregated streams: A-A… and B—B… (Figure 1). For intermediate frequency difference between the A and B tones, the grouping cues are ambiguous, and the percept is bistable.

Studies using such ambiguous ABA- tone sequences have shown that perceptual splitting of sound events with different acoustic features into different streams increases over time (Bregman, 1978; Anstis and Saida, 1985; Cusack et al., 2004). There is typically a period of time over which the probability of the segregated percept increases, starting from the initiation of a presentation (Bregman, 1978; Anstis and Saida, 1985) or a switch in the focus of attention (Cusack et al., 2004). In most of the reported experimental grouping paradigms, the initial probability of a split percept is zero, indicating that the first percept is always the grouped one (Anstis and Saida, 1985; Carlyon et al., 2001; Micheyl et al., 2005; Pressnitzer et al., 2008; Hupé and Pressnitzer, 2012; but see Deike et al., 2012, who show that stimuli for which perception is very strongly biased toward segregation produce an initial split percept). A similar phenomenon has been reported in the visual modality. When viewing ambiguous dynamic plaids constructed from two drifting gratings at intermediate speed and angle, observers have reported first experiencing coherent motion of a unified plaid pattern, even when, in the long term, their perception is biased toward transparent motion of the individual gratings in each of their component directions (Von Grünau and Dubé, 1993; Hupé and Rubin, 2003; Rubin and Hupé, 2004; Hupé and Pressnitzer, 2012). The probability of observers reporting a split perceptual organization over time can be quantified as a buildup function. This can be stated quantitatively in terms of a discrete valued perceptual state variable, *Z*(*t*), with a value of either 0 or 1 that may switch back and forth over time. *Z*(*t*) = 0 indicates a grouped perceptual organization at time *t* and *Z*(*t*) = 1 indicates a split perceptual organization; the buildup function is the probability that *Z*(*t*) = 1. Experimental evidence indicates that the initial percept is grouped, and then alternates (Pressnitzer and Hupé, 2006; Deike et al., 2012). We use this feature as an assumption and define the buildup function as the probability *Z*(*t*) = 1, given that the value of Z at time zero is 0, i. e., Pr{*Z*(*t*) = 1|*Z* (0) = 0}. The buildup function approaches a steady state value that equals the fraction of time that the split organization is dominant.

Such perceptual dynamics are of great interest in audition because they likely involve the same mechanisms that enable listeners to perceptually organize the individual sound sources in a complex auditory scene, a process known as stream segregation. This segregation, coupled with attention, is central to solving what is commonly referred to as the cocktail party problem (for a review, see Pressnitzer et al., 2011)—how do listeners follow a single speaker in a complex auditory environment against competing background noise? Various quantitative descriptions of the buildup function invoke proposed mechanisms of stream segregation. One theoretical explanation for the perceptual organizations observed with the ABA- stimuli is grouping by coactivation (for a review, see Carlyon, 2004). Neurophysiological investigations have found evidence that sound signals which excite the same population of neurons are grouped, whereas those that activate separate populations are perceived as coming from separate sources, that is, split. Based on these findings from short presentations (Micheyl et al., 2005; Pressnitzer et al., 2008; Bee et al., 2010), some propose that the buildup function reflects the accumulation of adaptation over seconds, or multi-second habituation. Over time, the neurons tuned to each tone frequency become more selective, and respond less to the other tone.

The accumulation-based account of the buildup function has produced neurometric models that can quantitatively predict the switch from the grouped to the split percept. These models nicely provide correspondence between neurophysiology and the buildup function found on short presentations, but they do not attempt to describe continuous alternations observed in other psychophysical paradigms over long presentations (Anstis and Saida, 1985; Pressnitzer and Hupé, 2006; Denham et al., 2012). Another well-reported feature of these psychophysical data is the absence of correlations between successive percept durations. Pressnitzer and Hupé (2005, 2006) demonstrate that ambiguous auditory and dynamic plaid stimuli display the same independence between successive durations as shown for other cases of perceptual bistability (Levelt, 1968; Rubin and Hupé, 2004). If buildup were necessarily an accumulative process, we would expect to see history dependence in the durations of successive percepts.

We bypass the mechanistic issue and show that the buildup function can be described well and quite generally by a statistical model that ignores accumulation over percepts. The gradual increase in probability of a split percept over time could reflect the dynamics of switching between percepts with independent random durations and a given initial state. The long-term dynamics of perceptual bistability consist of alternations between mutually exclusive percepts. The duration histogram of each percept has been well-fit by a gamma density, although log-normal or Weibull density functions can also be used (Pressnitzer and Hupé, 2006; Shpiro et al., 2009). We show that the short-term increase in probability of split percepts, observed when short trial perceptual time courses are averaged, could reflect the dominance duration distributions observed over long trials. To test this idea, we use the theoretical framework of an alternating renewal process (ARP). We use the fitted gamma densities for the dominance durations, without consideration for history dependence between successive durations, to account for the experimentally measured buildup function for a stimulus with ambiguous grouping, in models and experiments. The statistical model is general and based on the following underlying assumptions grounded in psychophysical evidence (Pressnitzer and Hupé, 2006; Hupé and Pressnitzer, 2012): (1) the perceptual state alternates back and forth between grouped and split; (2) the durations for these perceptual epochs are random, independent and stationary; and (3) the initial percept on for a given presentation is always the grouped percept. We implement this model using both Monte Carlo simulations as well as an analytical solution to the ARP model. The model covers a range of buildup functions: monotonic as well as multi-peaked and those exhibiting damped cyclic behavior.

In addition, we adapted existing neuro-mechanistic computational models frequently used to characterize perceptual bistability to produce buildup functions, in order to explore how different mechanisms of alternation affect buildup functions so produced, as well as the performance of our statistical model in describing them.

## Materials and methods

**Figure 2:**
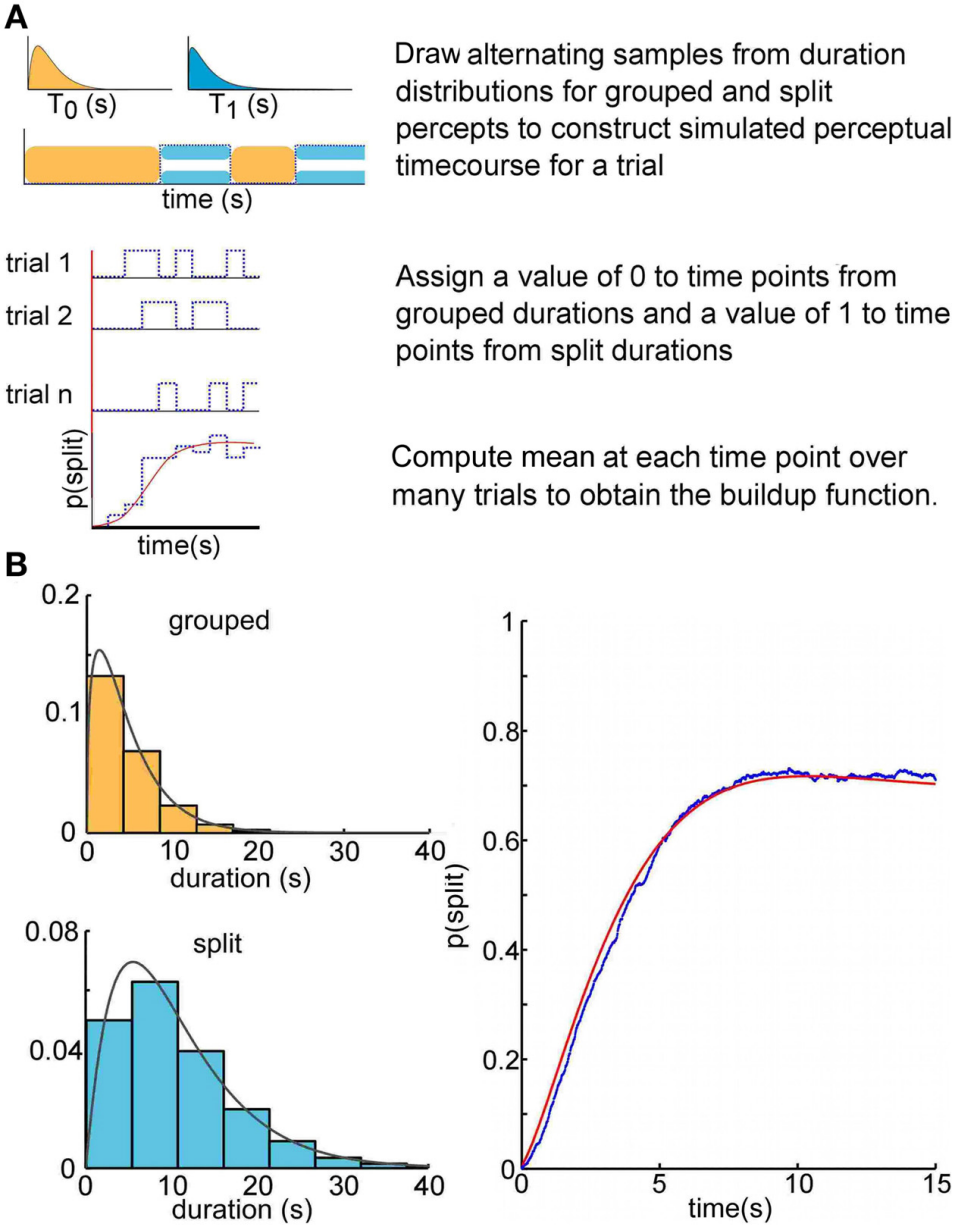
**Buildup as an alternating renewal process. (A)** Visualization of the alternating renewal process producing a buildup function. We used gamma probability density functions for duration distributions to construct Monte Carlo simulations. The simulated durations were drawn from gamma density functions with parameters α_0_ = 1.45, μ_0_ = 4.73 (grouped percept) and α_1_ = 2.08, μ_1_ = 10.65 (split percept), in the range of those typically reported in the psychophysical literature. The analytical solution to the alternating renewal process model, shown as a red solid curve (bottom), allows us to predict the buildup function solely from the distributions of dominance durations of each of the perceptual states. **(B)** Monte Carlo simulation computed buildup function of 1000 trials (blue) approaches the theoretical solution (red).

To compute a buildup function empirically, one averages over many trials the time course of a random binary state variable (see Figure 2A, blue lines). In our statistical model, the initial state (percept) is fixed, but the dwell time in this state is a random variable characterized by its probability density function. Subsequently the system switches randomly between two states, each of which has its own fixed duration distribution. This constitutes an ARP (for a review of renewal theory, see Cox, 1962).

We initially tested this theory in Monte Carlo simulations by simply constructing *in silico* perceptual time courses according to the above assumptions (see Figure 2A). For a given simulated trial time course, we draw alternating random samples from each of two distributions—one corresponding to the grouped state durations, and the other to the split state durations. These gamma distributions were specified randomly with parameters within the bounds [1,12]. These bounds were decided upon after visual inspection of many Monte Carlo simulated buildup functions, for sake of simplicity, and are typical for experimental data from perceptual bistability paradigms (Pastukhov et al., 2013; Huguet et al., 2014). We draw the first sample from the distribution corresponding to the grouped state, the second from that corresponding to the split state, and continue drawing samples from each distribution in alternation until the sum of all the durations exceeds the specified length of a trial. These trial durations were converted into discretized time courses by assigning a value of 0 or 1 to time intervals during which the state corresponds to a grouped or split percept, respectively. In Monte Carlo simulations, we produce an arbitrarily large (1000 trials) number of such time courses, and then take the average at each time point. This gives a relative frequency estimate of μ_*Z*_(*t*), i. e., the mean of *Z* (*t*).

We make explicit use of an analytical expression for the buildup function, by a method used in the closely related problems of Mortensen (1990), Kakubava (2008), and Stinchcombe et al. (2012). There are a number of advantages to characterizing the buildup function in this way. First, with an analytical solution relating the distributions of durations for grouped and split percepts to the buildup function, it is theoretically possible to interconvert between buildup functions and the statistics of the dominance durations for each percept. We have developed this solution into a statistical switching model to reconstruct the buildup function from four parameters—the parameters for the gamma densities for grouped and split percept durations. This theoretical solution coincides with the Monte Carlo simulation results (see Figure 2B). The analytical solution is convenient because it is computationally less expensive than iterative Monte Carlo simulations, and the solution is exact.

We wanted to test whether it was possible to recover the parameters for the long-term statistics of dominance durations from the buildup function. To estimate the gamma parameters from Monte Carlo generated buildup functions, we used the analytical solution (below) and searched for the 4 parameters that minimized the sum of the squared errors between the analytical and the Monte Carlo generated buildup function. We chose not to weight the different points in the fit by their standard error of the estimated probability for our computed buildup function, $\hat{p}$, as the highest value this could possibly take on would be $\frac{\sqrt{np\left(1-p\right)}}{n}=0.011$ for *n* = 500, *p* = 0.5 All simulations were implemented in MATLAB.

### Solving analytically for the alternating renewal process

In a stochastic, binary, switching process with independently distributed dwell times there are 2 random variables, *S*(*t*) and *Z*(*t*). *S*(*t*)is the random elapsed time since last switching into the state occupied at time *t*. The dichotomous random state variable, *Z*(*t*) = *i*, where *i* ϵ {0, 1} codes for the percept, grouped or split, respectively. We hypothesize that the experimentally observed perceptual switching process can be well described by an ARP (Cox, 1962).

We consider an ensemble of trials in which the states switch stochastically. For sake of simplicity, we specialize to the case of a fixed starting state across the population. Because our goal is only to find the buildup function, defined in this context as Pr{*Z* (*t*) = 1|*Z*(*t* = 0) = 0}, it is not necessary to derive the joint distribution for *Z* (*t*) and *S*(*t*).

We define the buildup function more specifically, in the context of our statistical model, as:

(1) Pr{Z(t)=1|Z(t=0)=0 ∩ S(t=0)=0}

For the sake of allowing for a special probability density function for the random duration of the first grouped percept, *T*^0^_0_, we first write the solution for $p_{1|1}\left(t\right)\overset{\text{def}}{=}\text{Pr}\left\{Z\left(t\right)=\;1|Z\left(t=0\right)=1\right\}$. This probability can be found by summing the probabilities of all the mutually exclusive ways the event {*Z*(*t*) = 1|*Z*(0) = 1} can occur (Mortensen, 1990; Kakubava, 2008; Stinchcombe et al., 2012). Calculating this probability in the time domain involves an infinite sum of terms, each of which is a convolution. However, we can obtain a simple analytical expression for ${\hat{p}}_{1|1}\left(\omega \right)$, the Fourier transform of *p*_1|1_ (*t*), i. e.:

(2) $${\hat{p}}_{1|1}\left(\omega \right)=\frac{{\hat{\tilde{F}}}_{T_{1}}\left(\omega \right)}{1-{\hat{f}}_{T_{0}}\left(\omega \right){\hat{f}}_{T_{1}}\left(\omega \right)}$$

The simple equation for ${\hat{p}}_{1|1}\left(\omega \right)$ obscures its origin. As one studies in calculus, for $\left|x\right|<1,\;\sum_{k=0}^{\infty }x^{k}=\frac{1}{1-x}$. Thus, the simple form of ${\hat{p}}_{1|1}\left(\omega \right)$ results from summation of the series ${\hat{p}}_{1|1}\left(\omega \right)={\hat{\tilde{F}}}_{T_{1}}\left(\omega \right)\sum_{k=0}^{\infty }{\left[{\hat{f}}_{T_{0}}\left(\omega \right){\hat{f}}_{T_{1}}\left(\omega \right)\right]}^{k},$ where *T*_0_ and *T*_1_ are the durations of the grouped and split percepts, respectively. The term in square brackets is the Fourier transform of the probability density function of the random variable *T*_0_ + *T*_1_, i.e., the random time spent in passing once through the sequence of *Z*-states (1, 0) before passing again into state *Z* = 1. Each term in the series for ${\hat{p}}_{1|1}\left(\omega \right)$ is the Fourier transform of a probability at time *t*. The first, ${\hat{\tilde{F}}}_{T_{1}}\left(\omega \right)$, is the Fourier transform of the probability that, given the state *Z* = 1 was entered at time 0, the dwell time before switching for the first time out of this state is greater than *t*. The kth term is of the probability that the sequence of *Z*-states (1, 0) is passed through exactly k times before entering state *Z* = 1 again at any time *t*′ < *t*, and that the transition out of state *Z* = 1 occurs at some dwell time greater than *t* − *t*'.

The term on the right-hand side of Equation (2) has a simple pole at ω = 0. Consequently, for computational purposes, it is convenient to write the inverse Fourier transform as:

(3) $$p_{\text{1|1}}\left(t\right)=\frac{1}{2\pi }\int_{-\infty }^{\infty }d\omega \frac{1}{i\omega }\left[{\hat{\tilde{F}}}_{T_{1}}\left(\omega \right)\frac{i\omega }{1-{\hat{f}}_{T_{0}}\left(\omega \right){\hat{f}}_{T_{1}}\left(\omega \right)}\right]e^{i\omega t}$$

where $\hat{f}(\omega )$ is the Fourier transform of *f*(*t*). The term in square brackets in the integrand has a limit of μ_*T*_1__/(μ_*T*_1__ + μ_*T*_0__) as ω approaches 0, and the 1/*i*ω coefficient of the term in square brackets is the Fourier transform of the integral operator. Therefore, *p*_1|1_ (*t*) can be written as:

(4) $$p_{\text{1|1}}\left(t\right)=\int_{0}^{t}dt\left\{\frac{1}{2\pi }\int_{-\infty }^{\infty }d\omega \left[{\hat{\tilde{F}}}_{T_{1}}\left(\omega \right)\frac{i\omega }{\text{1}-{\hat{f}}_{T_{0}}\left(\omega \right){\hat{f}}_{T_{1}}\left(\omega \right)}\right]e^{i\omega t}\right\}\text{​​​​​}$$

This function was called “availability,” A(t), by Pham-Gia and Turkkan (1999). Their time-domain solution is equivalent to our Equation (4). To find $p_{1|0}\overset{\text{def}}{=}Pr\left\{Z\left(t\right)=1|Z\left(0\right)=0\;\cap \;S\left(0\right)=0\right\}$, we time shift Equation (1) above by all possible durations of the initial state, *T*^0^_0_, weighted by density function of this state, *f*_*T*^0^_0__(*t*). This constitutes a convolution of the density function for initial duration with Equation (1):

(5) $$p_{\text{1|0}}\left(t\right)=\int_{0}^{t}f_{T_{0}^{0}}\left(s\right)p_{\text{1|1}}\left(t-s\right)ds$$

Thus, the solution can be given in the Fourier domain as:

(6) $${\hat{p}}_{\text{1|0}}\left(\omega \right)={\hat{f}}_{T_{0}^{0}}\left(\omega \right){\hat{p}}_{1|1}\left(\omega \right)$$

Using the simplifying assumption that *f*_*T*^0^_0__ (*t*) = *f*_*T*_0__ (*t*), that is, that the initial percept duration is from the same density function as all other *T*_0_, we find:

(7) $$p_{\text{1|0}}\left(t\right)=\int_{0}^{t}dt\;\left\{\frac{1}{2\pi }\int_{-\infty }^{\infty }\text{​}\text{​ }d\omega \left[{\hat{f}}_{T_{0}}\left(\omega \right){\hat{\tilde{F}}}_{T_{1}}\left(\omega \right)\frac{i\omega }{-{\hat{f}}_{T_{0}}\left(\omega \right){\hat{f}}_{T_{1}}\left(\omega \right)}\right]e^{i\omega t^{\prime }}\right\}$$

This equation states that *p*_1|0_ (*t*) can be obtained by taking the inverse Fourier transform of the term in square brackets, followed by integrating over time from 0 to *t*. We generally performed such computations numerically on a time axis from 0 to 40 s to ensure that the window for the discrete Fourier transform contained the function's approach to steady state, with 2^12^ sampling points (Bracewell, 2000).

Following previous research (Pressnitzer and Hupé, 2006; Shpiro et al., 2009), we used gamma density functions to generate and characterize state durations. That is:

(8) $$f\left(t;\mu ,\alpha \right)=\frac{\alpha }{\mu }{\left(\frac{\alpha t}{\mu }\right)}^{\alpha -1}\left(\frac{1}{\Gamma \left(\alpha \right)}\right)e^{-\frac{\alpha t}{\mu }}$$

where α is the shape parameter, and μ gives the mean duration (in many computing applications, the scale parameter $\theta =\frac{\mu }{\alpha }$ is used). In the special case that the dwell time densities are both gamma density functions with integer shape parameters, we can compute the buildup function *p*_1|0_ (*t*) entirely analytically. These solutions are the sum of exponential functions of time. For example, in the case of a common gamma density function with integer parameter, *n*, the solution is the weighted sum of 2(*n* − 1) complex exponential functions of time and one real exponential function of time. Each complex exponential sums with its complex conjugate partner to give *n* − 1 real functions of time. Thus, the overall solution is the sum of n terms. The *kth* term, which we call *g_k_*(*t*), is given by:

(9) $$g_{k}\left(t\right)=c_{k}\left\{1-\text{exp}\left(-\lambda_{k}t\right)\text{ sin}\left(2\pi f_{k}t+\theta_{k}\right)\right\},\text{for }k\text{=1},2,\ldots ,n,$$

where *c_k_* are constants, λ_*k*_ = (*n*/μ) [1 − cos(π*k*/*n*)], *f_k_* = (1/2π) (μ/*n*) sin (π*k*/*n*), and θ_*n*_ = 0.

**Figure 3:**
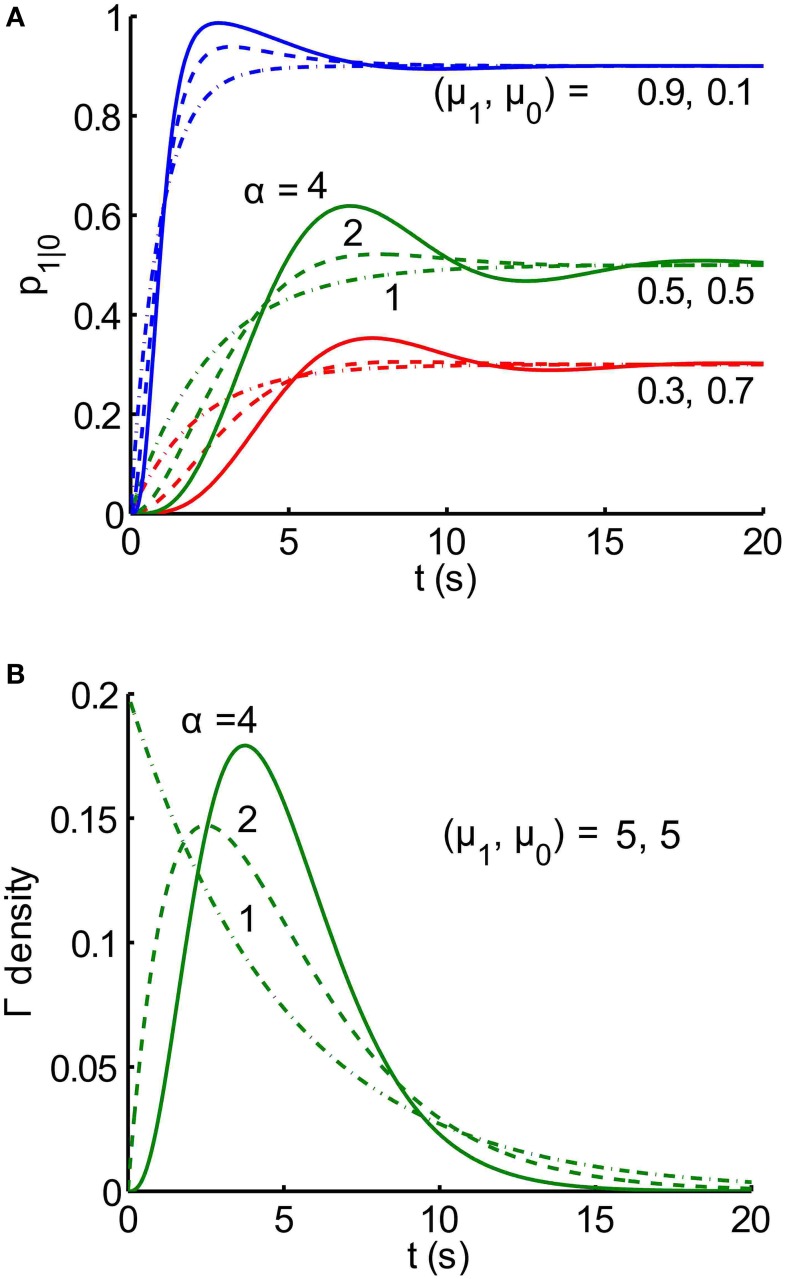
**Buildup functions from the analytical solution for an alternating renewal process. (A)** Families (red, green, blue) of analytical buildup functions [Equation (9)] produced by random, gamma-distributed dwell times, *T*_0_ and *T*_1_, in states *Z* = 0 and *Z* = 1, respectively. Each color corresponds to a different pair of mean dwell times, μ_1_ and μ_0_ for states *Z* = 0 and *Z* = 1, respectively, as labeled on the plots. Within each color, the shape parameter α takes on three different integer values *n*: 1 (dash-dot), 2 (dashed), and 4 (solid). Note that the steady-state asymptote for each buildup function is equal to μ_1_/(μ_1_ + μ_0_). Each buildup function is the sum of *n* exponentially damped sinusoids, given by Equation (9), with computed coefficients *c_k_* and phases θ_*k*_, where *k* = 1, …, *n*. For example, for the solid green curve (*n* = 4, and μ_1_ = μ_0_ = 5 s), the 4 temporal frequencies *f_k_* are 0.0900, 0.127, 0.090, 0 Hz, for *k* = 1, 2, 3, 4, respectively. The corresponding exponential decay rate constants λ_*k*_ are: 0.234, 0.800, 1.366, 1.60 s^−1^. Time constants, defined for the first three components, are given by the reciprocals of the rate constants: 11.1, 7.85, 11.1 s. **(B)** Gamma probability density functions *f_T_*(*t*) [Equation (8)] for random duration, *T*. The mean duration μ_*T*_ is equal to 5 s for all 3 densities, and the shape parameter α takes on 3 integer values *n*: 1 (dash-dot), 2 (dashed), and 4 (solid). As the shape parameter increases, probability is more concentrated around the mean value, μ_*T*_ = 5. In fact, lim_*n* → ∞_ *f_T_* (*t*) = δ (*t*-μ_*T*_).

Notice that the transient part of *g_k_*(*t*) is the product of 2 functions: the first is a decaying exponential function of time (*t*), and the second one is a sinusoidal function of *t*. The terms together can be thought of as a damped sinusoidal function of time under an exponential envelope. Examples of analytical buildup functions, *p*_1|0_ (*t*), are plotted in Figure 3; the legend includes the values of λ_*k*_, and *f_k_* for μ = 5, and *n* = 4.

### Population dynamics approach

A complete and more intuitive way of characterizing an ensemble (population) of trials, in which an ARP governs the switching of states, is to track both the *Z* (*t*) and *S* (*t*). For the sake of facilitating exposition, it is helpful to think of *S* (*t*) as the time from last switch (*age*) of a randomly drawn individual trial at time *t* (Karlin, 1958). In this analogy, switching state is accompanied by rejuvenation, in the sense that the individual's age is reset to zero. We take a population dynamics perspective in which there is, by convention, a single population including both *Z* = 0 and *Z* = 1, rather than separate populations for each. The state of an ensemble (population) is characterized at time *t* by its distribution over *s* and *z*, and this distribution is quantified by a probability density-mass function, ρ_*i*_ (*s*, *t*), defined as:

(10) $$\rho_{i}\left(s,t\right)ds\overset{\text{def}}{=}\text{Pr}\left\{S\left(t\right)\in \left(s,s+ds\right)\cap Z\left(t\right)=i\right\}$$

We are interested in the evolution of ρ_*i*_(*s*, *t*) starting from an initial state as members of the population age, randomly switch states and undergo rejuvenation. In this approach, the hazard function, *h_i_*(*s*), plays a central role. By definition, in the context of our problem, *h_i_*(*s*)*dt* = Pr{switch out of state *i* in (*t*, *t* + *dt*)|*Z*(*t*) = *i* ∩ *S*(*t*) = *s*}. The hazard function for state *i* is related to the probability density function for the dwell time in state *i*, by $h_{i}\left(s\right)=\frac{f_{i}\left(s\right)}{\int_{s}^{\infty }f_{i}\left(x\right)\;dx}$.

The pair of partial differential equations for ρ_*i*_(*s*, *t*) is derived from the principle of conservation of probability. Three essential ideas are: (a) In a time interval of width *dt*, there is only one way that probability can enter the interval *s*_0_ < *s* < *s*_1_, given *Z* (*t*) = *i*, and that is by individuals growing older and entering (*aging* into) the interval at *s*_0_. (b) One way of exiting the interval is by growing older and exiting (*aging* out of) the interval at *s*_1_. (c) A second way of exiting the interval is by switching out of state *i* at any age between *s*_0_ and *s*_1_, with a probability per unit time, per unit age, given by *h_i_*(*s*) ρ_*i*_ (*s*, *t*). The ideas (a—c) give rise to the coupled partial differential equations:

(11) $$\frac{\partial \rho_{0}}{\partial t}\left(s,t\right)=-\frac{\partial }{\partial s}\left(\rho_{0}\left(s,t\right)\right)-h_{0}\left(s\right)\;\rho_{0}\left(s,t\right)$$

(12) $$\frac{\partial \rho_{1}}{\partial t}\left(s,t\right)=-\frac{\partial }{\partial s}\left(\rho_{1}\left(s,t\right)\right)-h_{1}\left(s\right)\;\rho_{1}\left(s,t\right)$$

A fourth essential idea is: (d) When a member of the ensemble switches out of one state (*Z*ϵ {0,1}) at age *s*, it appears instantaneously in the other state at age *s* = 0}. Idea (d) gives rise to the boundary conditions:

(13) $$\rho_{0}\left(0,t\right)=\;\int_{0}^{\infty }h_{1}\left(s\right)\;\rho_{1}\left(s,t\right)ds$$

(14) $$\rho_{1}\left(0,t\right)=\;\int_{0}^{\infty }h_{0}\left(s\right)\;\rho_{0}\left(s,t\right)ds$$

The solution of the system in Equations (10) and (11), with given initial conditions, gives the joint distribution for the random state *Z* (*t*) and random age *S* (*t*). The probability that a member of the ensemble is in state *i* at time *t* is given by integrating over age, i.e., Pr{*Z*(*t*) = *i*} = ∫^∞^_0_ ρ_*i*_(*s*, *t*) ds. The problem we solved above by more elementary means corresponds to the initial conditions ρ_1_ (*s*, 0) = δ (*s*), and ρ_0_ (*s*, 0) = 0. In other words, all members of the ensemble are in state *Z* = 1 with age s = 0 at time *t* = 0. We derived our analytical buildup function by solving the pair of linear partial differential equations and boundary conditions Equations (11)–(14) for the joint probability density-mass function for age and perceptual state defined in Equation (10). At an intermediate stage, the solution was in the form of a coupled pair of integral equations, each involving a convolution. We solved these by taking the Fourier transform of each convolution equation and then solving a linear pair of algebraic equations in the two density-mass functions. Integrating these over the continuous age variable, *s*, gave us the buildup function as defined in Equation (1).

### Competition model simulations

**Figure 4:**
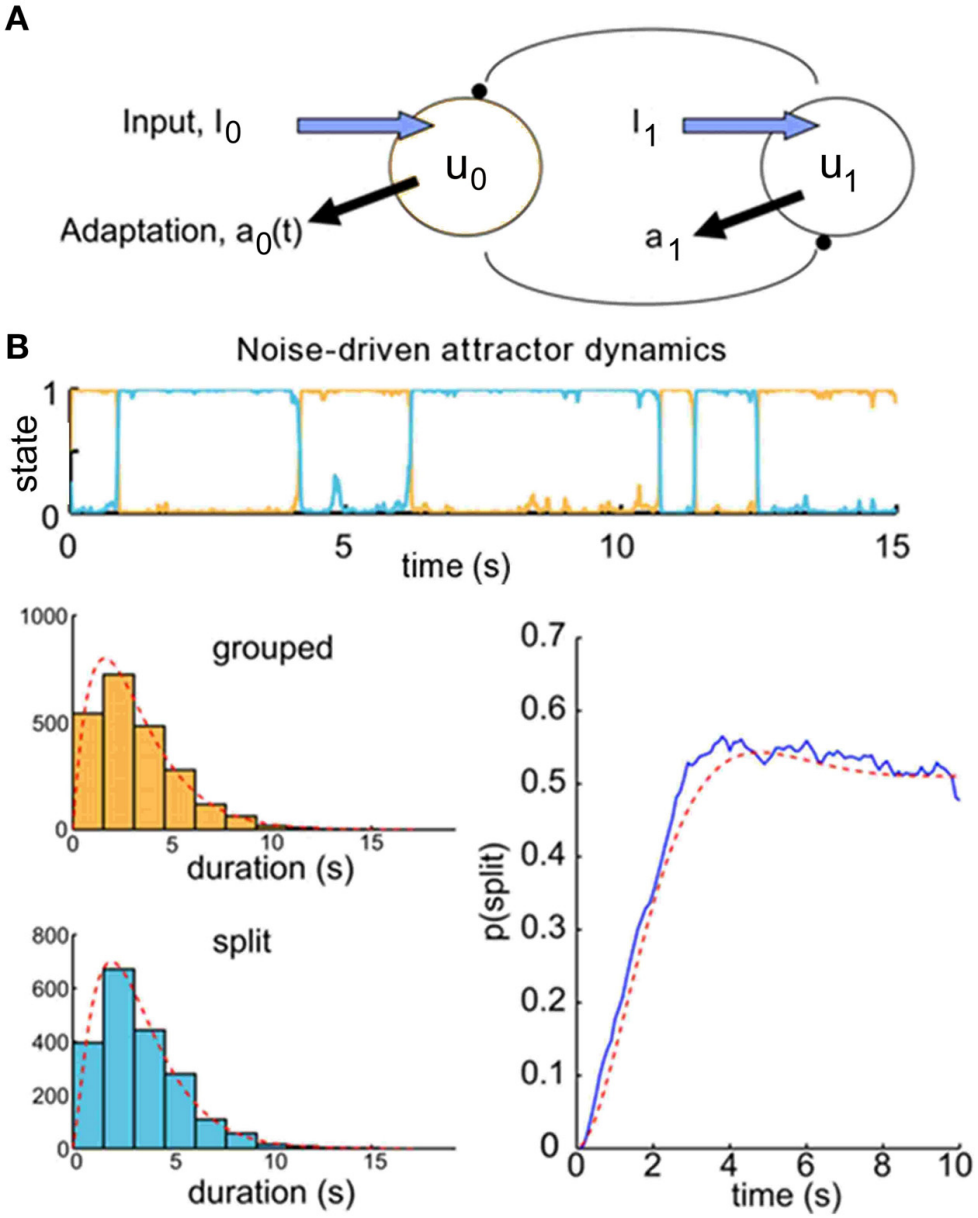
**Buildup functions from neuronal competition model with attractor dynamics**. **(A)** Mutual inhibition population firing rate model producing buildup. We choose initial conditions to ensure that the population representing the grouped percept, u_0_, is always dominant at the beginning of a given trial time course. **(B)** Competition model simulation results for parameters that produce attractor dynamics with noise-driven switching: γ = 0.1 and σ = 0.12. Correlations between successive durations are low (*r* = 0.11). Top, population activity time course for one 20-s trial. We simulated 500 trials to produce the buildup function, lower right (blue). Histograms of the dominance durations, with maximum likelihood estimated gamma density parameters (α_0_ = 2.02, μ_0_ = 3.17; α_1_ = 2.40, μ_1_ = 3.34) and the associated density functions (gray), are shown in the lower left. These parameters allow us to compute analytically the resulting buildup function for an alternating renewal process (red). The buildup function looks similar to those reported in the psychophysical literature, and the statistical model's prediction is good (*R*^2^ = 97%).

We use a competition model as a test-bed for the theory of the ARP for different dynamical regimes of perceptual alternation—in particular, for noise-driven switches, for which correlation between successive dominance durations is low, and for adaptation-driven switching, in which correlation is high. We chose to use existing observer models for perceptual bistability to produce buildup functions to see if we could relate these to the underlying dominance durations using renewal theory. Previous investigations (Wilson and Cowan, 1972; Wilson, 2003; Shpiro et al., 2009; Laing et al., 2010; Pastukhov et al., 2013) have used population firing rate models with competition architecture to model perceptual bistability. In these pseudoneuronal mutual inhibition models, there are separate populations whose firing rates represent the perceptual strength of each interpretation of the stimulus. They make inhibitory connections onto one another, so the population with the highest firing rate typically dominates the other (Figure 4A). These models were originally developed to describe binocular rivalry, but have also been used to account for the psychophysical results of experiments with ambiguous grouping–namely, moving plaids with coherent/transparent motion (Shpiro et al., 2009; Pastukhov et al., 2013) and triplets with streaming integration/segregation (Mill et al., 2013).

In competition models, the relative firing rates of the two populations are taken to produce the simulated observer's perceptual reports. The population with the higher firing rate corresponds to the dominant percept. Because the two populations mutually inhibit each other, in most cases only one population is active at any given time. In addition, each population undergoes adaptation in response to its own firing rate. The alternation of dominance epochs between the two populations can be driven by two mechanisms. If adaptation is strong enough, then the activity of the dominant population will decay over time, while the suppressed population recovers from any prior adaptation. This leads to periodic alternations between dominance states with noisy oscillator dynamics. However, if adaptation is weak, the system will display attractor dynamics, in which alternations are driven by noise in the externally applied inputs. The brain appears to be a very noisy system, with random fluctuations occurring at multiple scales such as vesicular release and spiking variability. The competition models with attractor dynamics, in which switching between dominance epochs is driven by noise, appear to be more consistent with the statistics of dominance durations observed in psychophysical experiments (Shpiro et al., 2009), and we exploit the fact that the models operating within this regime produce buildup functions that are consistent with those measured experimentally.

Competition model simulations followed the procedures reported previously in Shpiro et al. (2009) for population firing rate model with spike frequency adaptation. Specifically:

(15) $$\{\begin{array}{l} {\dot{u}}_{0}=-u_{0}+f\left(-\beta u_{1}-\gamma a_{0}+I_{0}+n_{0}\right)\\ \tau_{a}{\dot{a}}_{0}=-a_{0}+u_{0}\\ {\dot{n}}_{0}=-\frac{n_{0}}{\tau_{n}}+\sigma \sqrt{\frac{2}{\tau_{n}}}\eta \left(t\right)\\ {\dot{u}}_{1}=-u_{1}+f\left(-\beta u_{0}-\gamma a_{1}+I_{1}+n_{1}\right)\\ \tau_{a}{\dot{a}}_{1}=-a_{1}+u_{1}\\ {\dot{n}}_{1}=-\frac{n_{1}}{\tau_{n}}+\sigma \sqrt{\frac{2}{\tau_{n}}}\eta \left(t\right) \end{array}$$

The variable *u*_0_ corresponds to the short-time averaged firing rate of the population representing the “grouped” perceptual state, and *u*_1_ to the firing rate of the population representing the “split” perceptual state. The variables *a*_0_ and *a*_1_ represent the spike-frequency adaptation. Parameter γ controls the strength of the adaptation, and β controls the strength of suppression from the competing population. *I*_0_ and *I*_1_ are the external inputs driving the two populations, and *n*_0_ and *n*_1_ are independent Ornstein-Uhlenback noise generators with mean zero and variance σ, and a timescale of τ_*n*_. The input-output function used was a sigmoid, with *f*(*x*) = 1/[1 + exp((*x* − θ)/*k*)].

The simulation was carried out in non-dimensionalized time, with the convention that one unit of time corresponds to 10 ms. Time constants given in simulation time units were τ_*a*_ = 200, τ_*n*_ = 10. The following parameter values are used: *k* = 0.1, θ = 0, β = 1. All simulations used the same input strength: *I*_0_ = *I*_1_ = 0.6. According to our computations, the border between attractor and oscillator dynamics (noise free case) for this strength of external input lies in the range γ = 0.45 to γ = 0.5 although the estimate reported in Shpiro et al. (2009) was close to γ = 0.25. For attractor dynamics, we used the parameter values: γ = 0.1 and σ = 0.12. For dynamics near the border of attractor and oscillator dynamics (but within the attractor regime), we set γ = 0.4 and σ = 0.09. For full oscillator dynamics, we set γ = 0.7 and σ = 0.06. The value of σ was scaled in relation to the integration time step by $1/\sqrt{dt}$ to keep specified variance per unit time. Simulations were implemented in MATLAB using forward Euler integration with a time step of 0.1 (1 ms in dimensioned time).

For each combination of parameter values, we simulated 500 trials of length 20 s with initial conditions *u*_1_(0), *a*_0_(0), *a*_1_(0), *n*_0_(0), *n*_1_(0) = 0 and *u*_0_(0) = 0.5; this initial condition for *u*_0_, guarantees that at the beginning of each simulated trial, the first population to become dominant was always that corresponding to the grouped percept. Simulated experimental buildup curves were constructed by computing the average for each time point across trials of the binary time course *u*_1_ > *u*_0_.

We obtained dominance durations by finding the times of switching {*t_s_*}, i.e., when *u*_0_(*t*) = *u*_1_(*t*). To test the application of the ARP model, we viewed dominance durations of each state from the short simulated trials as samples from underlying gamma distributions. Because our competition model simulations generated trials that were only 20 s long, a large proportion of these durations were truncated by the end of the trial. We estimated gamma parameters α and θ that maximized the likelihood of the complete as well as the right-censored dominance durations for each model perceptual state. Using the samples of dominance durations obtained for each population (over 1000 durations for each population with each parameter set), we fitted gamma densities using maximum likelihood estimation. We compared the simulated empirical buildup functions, using 2^12^ sample points, with those predicted under our statistical model for these fitted gamma parameters by computing *R*^2^, the coefficient of determination.

## Results

### Monte carlo simulated and analytically computed buildup functions agree

We propose that the buildup function arises from a system that alternates between two states from a known starting state, and that the dwell times in each state are independent with durations drawn from two probability density functions. Using renewal theory, we found an analytical solution that relates the buildup function to the density functions describing the state durations. We also performed Monte Carlo simulations to generate random samples from two gamma density functions with parameters α and μ [Equation (8)], construct simulated trials from these samples, and compute buildup functions (Figure 2). The statistical model uses the 4 parameters of the duration density functions to make a prediction for the buildup function under an ARP, and the Monte Carlo simulated buildup functions converge with this prediction. Given a pair of gamma density parameters describing the dwell times in each state, that is, grouped or split perceptual organization, we can generate an accurate prediction of the buildup function produced under an ARP.

An exploration of the relationship between gamma density parameters and buildup functions shows that higher shape parameters α produce buildup functions with more damped cyclic behavior (Figure 3). This is because a higher shape parameter results in probability being more focused around the mean, meaning the durations are less variable. If the durations were fixed, i. e. if the densities for durations were delta functions, then every trial would proceed deterministically and look exactly like the buildup function, which would be binary. It is only the variability in switch times that makes the buildup function appear smooth.

### Buildup functions from competition model dynamics are well described by an alternating renewal process, but quantitative accuracy is lower when correlations are higher

We used pseudo-neuronal competition models to produce experimental time courses similar to those reported in the psychophysical literature on ABA- tone sequences. Our statistical model makes three assumptions describing an ARP: the initial state is always the same, the state alternates back and forth, and the state durations are random, independent and stationary. By setting the initial conditions to ensure that the neuronal population representing the grouped percept was always active first, we were able to satisfy the assumption that the initial perceptual state is fixed. The competition models also satisfy the assumption of alternation between perceptual states of grouped and split. However, the state durations are not necessarily independent or stationary—depending on the dynamical regime in which the competition model is operating, attractor or oscillator, there can be significant history dependence between state durations.

The difference between oscillator and attractor dynamics in these competition models is most simply understood by observing how the system would behave without noise (Moreno-Bote et al., 2007; Shpiro et al., 2009). For oscillator dynamics, adaptation would cause the dominant population to reduce its activity over time, reducing the inhibition on the suppressed population, allowing it to become active. In a noiseless system, stable fixed points in the system appear and disappear over time, and alternations will occur deterministically with a constant period. Noise in such a system will affect the distribution of dominance durations for each state, but is not required for switching. Conversely, attractor dynamics occurs when a system has multiple stable states at the same time. In the absence of noise, the initial conditions determine which state becomes active, and the system behaves in a winner-take-all fashion. That is, the population that becomes dominant first is permanently active, and the other population is permanently suppressed. However, injecting noise into such a system can cause switches from one stable state into another. In this case, the switching between perceptual dominance states is caused by the noise itself.

**Figure 5:**
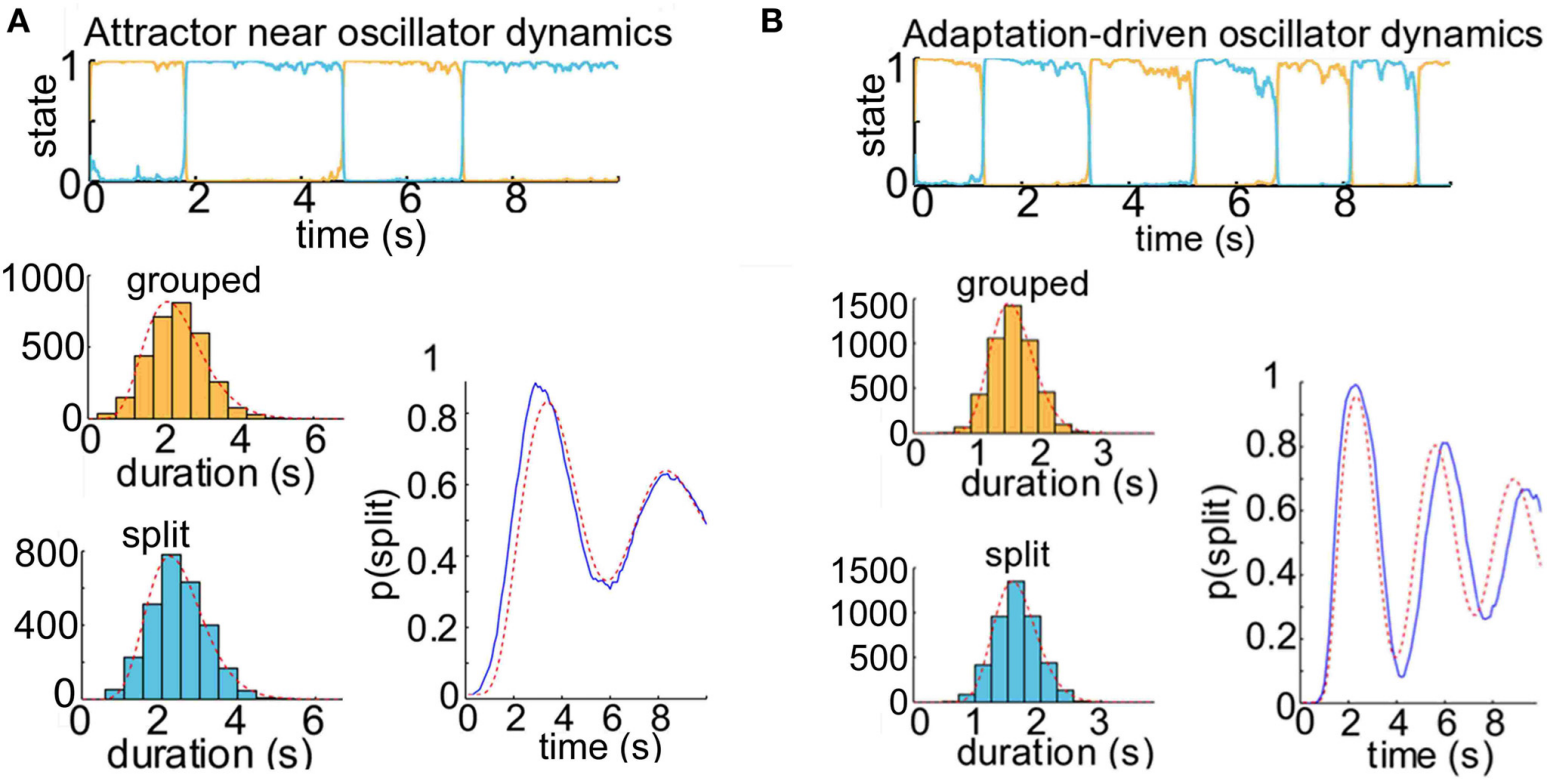
**Competition model simulation results for model parameters near or past the border of noisy oscillator dynamics**. When adaptation is a moderate or strong factor in our simulations of the neuronal competition between grouped and split percepts, the buildup function displays damped cyclic behavior. **(A)** Attractor dynamics near border with oscillator dynamics for neuronal competition model. With parameters γ = 0.4 and σ = 0.09, this model would not show alternations between states in the absence of noise, but adaptation is still a significant factor in facilitating switches. The buildup function displays damped cyclic behavior, and there are correlations between successive dominance durations (*r* ≈ 0.25). The alternating renewal process model with parameters α_0_ = 9.06, μ_0_ = 2.38; α_1_ = 11.34, μ_1_ = 2.48 provides a good estimate of the buildup function from the distribution of dominance durations (*R*^2^ = 91%), but the prediction is not as strong as when the parameters are further from the border with oscillation dynamics (see Figure 4). **(B)** Oscillator dynamics with neuronal competition model parameters γ = 0.7 and σ = 0.06. Correlations between successive durations are high (*r* ≈ 0.30). The dominance durations are much more regularly timed than those produced under attractor dynamics, reflecting the clock-like cycling of the underlying oscillator (the period of the noise-free oscillator is 2.2 s). These oscillations are dramatically present in the average over 500 simulated trials, lower right (blue). The maximum likelihood estimated gamma density functions are shown in the lower left (gray), and the analytically computed buildup function for an alternating renewal process with those gamma parameters (α_0_ = 21.51, μ_0_ = 1.60; α_1_ = 23.04, μ_1_ = 1.65) is shown in the lower right (red). The fit between the analytical solution and the trial average is much weaker (*R*^2^ = 77%) than for the attractor dynamics as in Figure 4.

When the switches between perceptual states are driven by noise, as under attractor dynamics, correlations between successive dominance durations tend to be low (*r*≈ 0.11). An example trial time course is shown in Figure 4B, top. To obtain a buildup function from the simulated competition dynamics we identify the split state, *Z*(*t*) = 1, according to the condition, *u*_1_(*t*) > *u*_0_(*t*), then by averaging *Z*(*t*) over many trials we obtain an empirical buildup function. We fitted gamma parameters to the dominance durations, and used these parameters to generate analytical buildup functions using the ARP model (Figure 4B, bottom). The predicted buildup function for an ARP with the underlying duration distributions with those fitted gamma parameters very strongly fits (*R*^2^ = 97%) the buildup function obtained empirically by averaging over many trials for this case with weak adaptation, γ = 0.1. When the adaptation strength, γ, is increased adequately the competition dynamics transition from noise-driven attractor mode to noisy oscillator mode, correlations increase, and the buildup function takes on a damped cyclic behavior. For near-oscillator dynamics (γ increased from 0.1 to 0.4, see Materials and Methods), the damped cyclic behavior is apparent and the correlation between successive percept durations increases from 0.11 to 0.25 (Figure 5A). In the oscillator regime (γ = 0.7, Figure 5B), the buildup function is strongly damped cyclic and the correlation coefficient is higher still, *r* ≈ 0.30. With these increases in correlations as γ increases, the quantitative accuracy of the ARP in describing the buildup function decreases, from *R*^2^ = 91 to 77% for γ = 0.4 to 0.7, respectively. Although the ARP provides a strong quantitative prediction of the buildup function for data with weak correlations, it loses quantitative accuracy for duration distributions that are less independent.

### Damped cyclic buildup functions can arise from competition in or near the oscillator regime

It has previously been found that noisy oscillator dynamics are not entirely consistent with a number of statistical features of the dominance durations reported in psychophysical experiments (Shpiro et al., 2009; Pastukhov et al., 2013). The mean and coefficient of variation of dominance durations for noisy oscillator dynamics when alternations are primarily adaptation-driven do not fall within the range of those observed for perceptual reports of ambiguous visual displays. Furthermore, when adaptation drives alternations in the dominance of population activity, we observe moderate and significant correlations between successive percepts. On the other hand, data from the psychophysical literature suggests that the durations of subsequent percepts are only weakly correlated, if at all (Levelt, 1968; Rubin and Hupé, 2004; Pressnitzer and Hupé, 2005, 2006).

We examined buildup under both oscillator and attractor dynamics in order to determine whether we could find correspondence between the buildup functions produced by competition models and those reported in the psychophysical literature. Previously reported psychophysical data indicate that the buildup function is typically monotonic. To our knowledge, no psychophysical experiments using an ABA- stimulus have shown a buildup function time course with a damped oscillatory approach to steady state, although Anstis and Saida (1985), using a two-tone stimulus, present non-monotonic psychometric functions on reports of temporal coherence that appear to be damped cyclic. Buildup functions produced with moderate adaptation in our competition model (Figure 5) display damped cyclic behavior, even when the mechanism of alternation is noise-driven attractor dynamics (Figure 5A). These buildup functions are derived from the model's perceptual time courses for which there are significant correlations between successive percept durations, such that the duration of the present perceptual state depends on the cumulative history of previous percepts. Although the correlation coefficient between durations of subsequent percepts was substantial (*r* ≈ 0.25), the fit to the buildup function by finding the parameters of density functions for long-term percept durations was strong (*R*^2^ = 91%). The gamma density functions describing dominance durations from competition models operating in or near the oscillator regime with moderate noise look more like delta functions—the dominance durations are not highly variable, and so switches are more likely to occur at similar times on different trials. This is why such gamma density functions produce buildup functions that look damped cyclic. While such buildup functions have not typically been reported for stimuli with ambiguous grouping cues, Borsellino et al. (1972) report that subjects who are fast switchers when viewing stimuli such as the Necker cube have more regular distributions of switch times, whereas slow switchers demonstrate more variability in percept durations; if this relationship holds true for plaid and auditory triplet stimuli, we could speculate that fast switchers may exhibit non-monotonic buildup functions.

### Inference of ARP parameters from the buildup function

**Figure 6:**
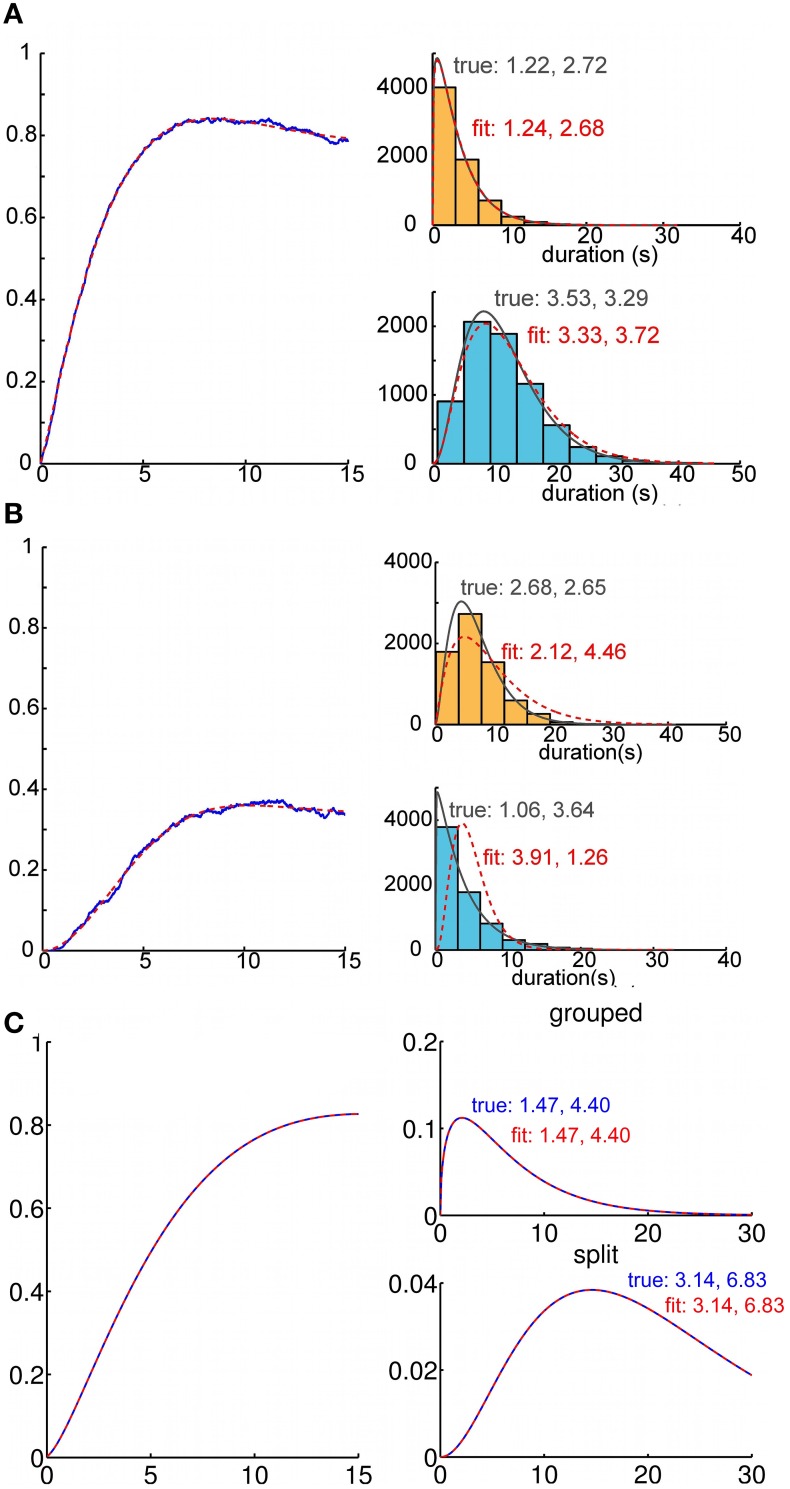
**Recovery of parameters for duration distributions from analytic buildup functions**. Recovery of percept duration distributions from a buildup function. We fitted parameters for the analytic buildup function by minimizing the squared error between the original and the fitted buildup function. This allows us to estimate the parameters of the underlying gamma density functions for percept durations. **(A)** Recovery of generating gamma density functions from buildup functions that were computed via Monte Carlo simulation (1000 trials). Usually the density functions corresponding to the fitted buildup function were a good match for those that had generated the sample buildup function. **(B)** Because the fitting process was very sensitive to noise, the density functions obtained from the fitted buildup function occasionally differed from the generating duration distributions. **(C)** From buildup functions that were computed analytically using our model, we always recovered the parameters of the gamma densities (i.e., α_0_ = 1.47, μ_0_ = 6.47; α_1_ = 3.14, μ_1_ = 21.45 for this buildup function) that had generated those buildup functions.

One might reasonably enquire whether ARP model parameters can be inferred based solely on an empirical buildup function (Figure 6). We studied this question in two ways. First, we generated empirical buildup functions by Monte Carlo simulations in which the durations of “perceptual states” were indeed generated by an ARP with parameters of our choice. Then, proceeding as if we did not know the parameters, we searched parameter space to minimize the sum of squared errors between the empirical buildup function and the theoretical one generated at various points in parameter space. We always found ARP parameters that provided a good fit to the empirical buildup function. Usually, we were also able to obtain accurate estimates of the generating gamma density functions (Figure 6A). However, this method did not consistently recover the correct parameter values (Figure 6B); it appears that the trial averaged buildup function itself does not constrain particularly well the parameters of the gamma density functions that describe the state durations on individual trials.

In a real experimental setting, even if buildup function were generated by an ARP, the “true underlying parameters” may be difficult to estimate accurately. Nevertheless, we conducted a second test in which we asked how well the original parameters in the generative model could be recovered by fitting the buildup function in the time domain. Although we did not conduct an exhaustive study of this type, our experiments suggested that the only limitation to parameter recovery was inevitable noise in the empirical buildup function. In other words, if the exact theoretical buildup function is provided as input data, the original parameters are recovered essentially exactly (Figure 6C).

## Discussion

During presentations of ambiguous stimuli subjects may perceive switching between integration and segregation. For short presentations (i. e., 10 s trials, as in Micheyl et al., 2005; Pressnitzer et al., 2008), there may be only one or a few switches after the initial percept—in the van Noorden ABA- paradigm discussed in this paper, and for visual plaids, the initial percept is typically integration (Hupé and Pressnitzer, 2012)—but for long presentations (i.e., 4 min trials as in Pressnitzer and Hupé, 2006; Denham et al., 2010), haphazard alternations typically occur.

We introduced and explored a statistical model for the buildup function, the probability of segregation versus time. Our model accounts for the buildup function as the mean over trials in the short presentation case, or the switch-triggered average time course for long runs (see Section “Switch-Triggered Buildup Function,” below), in terms of an ARP. Evidence accumulation or adaptation is not involved in our statistical dynamics model; the dynamics of buildup alone do not implicate an accumulative process. By definition a renewal process has no memory about the duration of the preceding percept; there are no correlations between successive percepts, consistent with reports from behavioral experiments (Pressnitzer and Hupé, 2006). The model contains no explicit description for an accumulative or adaptive mechanism.

The buildup function for our ARP model can be computed with Monte-Carlo simulations or it can be evaluated with the analytical solution (in terms of Fourier transforms) of the partial differential equations for the probability mass functions Equations (9) and (10) in the two states. In this direct framework, one assumes that the duration distributions for the two percepts are known, under stationary switching, say, for long runs. For our case studies, we assume gamma distributions, as they are often applied and fitted to behavioral data for bistable perceptual dynamics. To restate, the model enables one to understand and predict from the stationary statistics the system's transient behavior—the buildup function from a specified initial state. We applied the ARP model to predict buildup functions for neuronal-like competition models. The predictions compared well with the simulated empirical buildup functions for examples of monotonic buildup (as typically reported from experiments) as well as, to a lesser degree, non-monotonic (damped cyclic) buildup time courses—however, when perceptual state dwell times violate the model's assumption of independence, the prediction loses quantitative accuracy. Adaptation-driven switching is inconsistent with previous psychophysical reports, and the buildup functions obtained from neuronal simulations with strong adaptation and percept-to-percept correlations are unlike those reported in the literature. The ARP does describe well those neuronal models that produce perceptual state durations with statistics consistent with those from psychophysical experiments.

It is worth considering that bistable perception is a special case that exposes features of ordinary perception that frequently go unnoticed. In trying to interpret noisy sensory signals, competition between alternative perceptual states is constantly on-going, but usually the odds in favor of one interpretation are overwhelming (Pressnitzer et al., 2011)—particularly when attention is engaged to boost one signal and suppress all others. The cocktail party problem involves segregation and attention. Our model could trivially describe the stability of segregation when the percept durations in the segregated state are vastly longer than the durations in others, so that the durations of the unheard percepts were essentially always zero. The model also describes how the transient dynamics of buildup are linked to the stationary dynamics of random switching between alternative perceptual states when the stimulus is ambiguous.

### Relationship between steady-state and transient behavior

If the ARP model provides an accurate statistical description of experimentally measured buildup functions, the entire time course of the buildup function could be computed accurately from short-trial data that records only a few switches before a trial ends, even if that time is well before the buildup function reaches a steady state. Allowing for a special probability density function for the first percept, the only requirement for accurate reconstruction is the observations of a large enough minimal set of outcomes. In terms of the perceptual state variable *Z*, the minimal set of *Z* sequences (in which a switch out of the last percept in the sequence has not yet occurred at termination time) consists of {(0), (0, 1), (0, 1, 0), (0, 1, 0, 1)}. A large enough set of samples including these outcomes allows an accurate estimate of three duration density functions in the ARP model for: the first segregated percept; the integrated percept; and the (non-initial) segregated percept. In the context of the ARP model, no additional observations are required to predict the entire time course of the buildup function.

In a similar vein concerning predictive power, the ARP model enables one to understand and predict, from the stationary statistics, the transient time course of the buildup function: the temporal evolution, starting at time zero, of the probability of segregation, onward as that probability approaches its steady state. From a dynamical systems point of view, it may seem surprising to predict transients from knowledge of a steady state only. In our case of a two-state switching system with renewal, the two dwell time distributions contain all the information that is needed, just as long as the duration of the first percept is not special. If it is, then the steady-state statistics still characterize a relative of the buildup function that is obtained by defining time zero in each trial as the instant at which the switch out of the first state occurs.

### Switch-triggered buildup function

Although our model can account for experimental data in which the initial percept duration distribution is different from the steady state, this issue can be circumvented by a different averaging technique, similar to those commonly used in neurophysiological studies of several sensory modalities. In this approach, one constructs a steady-state buildup function by averaging over time points aligned by switches into the grouped percept, thereby producing a buildup function from a single long trial. Discarding the first and second percept durations, we can construct buildup functions by estimating the probability over time for the split percept based on an event-triggered average aligned to each switch into the grouped percept. This method produces a buildup function for the steady state of alternations, the probability of perceiving the split organization not just from the beginning of the trial but rather from the beginning of any grouped percept over the course of a long presentation. Similarly, if one constructs a buildup function for the steady state of switching into the grouped percept, there would be two buildup functions available for analysis. A statistical advantage in parameter estimation from laboratory data might be afforded by measuring two buildup functions. The value for analysis of having two buildup functions could be realized also in cases of bistable dynamics, such as in binocular rivalry, when the initial percept could differ across trials.

### What if the first percept is special?

For ambiguous stimuli, the time until the first perceptual switch can be on average notably longer than subsequent durations of the same percept (Pressnitzer and Hupé, 2006); this feature has been dubbed “inertia” (Hupé and Pressnitzer, 2012). The distribution of initial grouped percept durations would then be different from other grouped percepts. If recorded trials are short, say, containing one or fewer switches from integrated to the segregated percept, the distribution of initial switch time and the one just afterwards would be sufficient to describe a renewal process accounting for the very early phase of buildup. But these data would not likely suffice for predicting the steady state behavior. If trials are longer with many alternations, however, one could distinguish between initial and subsequent grouped percept durations. Our theoretical model can incorporate both the steady state and initial percept distributions. However, in the model/parameter identification problem, this would introduce a third duration distribution, and increase the number of parameters to 6. For simplicity's sake, we have only shown the 4-parameter model, which assumes that the initial percept duration is drawn from the same distribution as other grouped percept durations.

It is worth noting that there are circumstances in which the first percept is not longer than subsequent integrated percepts, such as when buildup resets after a switch in attention (Denham et al., 2010). Stationary distributions might be appropriate for such circumstances. Furthermore, our model is agnostic as to the mechanism by which the first percept is determined—possible underlying mechanisms have been thoroughly discussed in Hupé and Pressnitzer (2012). Our model functions as a statistical description of how probabilities evolve with random independent switching from a particular initial state.

### Mechanistic and computational models

An important component of a physiological model that accounts for the perceptual dynamics underlying the buildup function would be some description of the sensory coding mechanisms that underlie the formation of perceptual organization, as a start. Such a model would not necessarily account for the stochastic switching. The specific mechanisms for switching in human stream segregation may be complex; Kondo and Kashino (2009) find that feedforward and feedback processes in a thalamocortical loop might be differentially engaged for switches into and out of the perceptual organization that is strongest. The renewal process model is agnostic as to the specific mechanism by which states are found and alternations occur. We have used existing competition models for the sake of illustration and as a computational test-bed. Similar competition-like processes have been used to explain the alternations observed in ambiguous motion (Pastukhov et al., 2013) and stream segregation (Mill et al., 2013) experiments. A better understanding of the characteristics of the neural populations on the encoding side of perceptual organization could enable us to more accurately model the psychophysical data.

Previous computational approaches to describing the buildup function (Micheyl et al., 2005; Pressnitzer et al., 2008) have pointed to the accumulation of adaptation as a critical feature for the increase over time in the probability of a split percept. Multi-second habituation in the auditory periphery (Pressnitzer et al., 2008) can predict the buildup function obtained through psychophysics. It may therefore be surprising that the ARP, which seemingly lacks any notion of adaptation, can provide a good statistical model for buildup. However, we believe that previous approaches and our own can be reconciled, and may even be complementary. The choice of gamma densities to generate dominance durations implicitly invokes adaptation (Wilbur and Rinzel, 1982) for the following reason. In an ARP one can define a hazard function, the probability per unit time of a switch out of state *Z_i_*, given an elapsed time *s* since entering this state. The hazard function for a gamma density, in contrast to an exponential distribution, is dependent on the elapsed time since switching into each state. Therefore, there is a within-epoch memory which might be thought of as a local adaptation However, it is important to note the distinction between this dependence on time since last switch, *s*, and the clock time dependence of cumulative habituation, a memory that spans multiple epochs, or the dependence on neuronal activity of global adaptation. Rubin and Hupé (2004) have found stability over time of the statistics of durations, indicating that there is no cumulative adaptation.

Our statistical model for buildup is agnostic on the mechanism of switching, and this is a strength in some important respects. We take observations from the experimental literature as our starting point—that perception alternates randomly between grouped and split (Anstis and Saida, 1985; Pressnitzer and Hupé, 2006; Denham et al., 2012), that the durations in each state are random and independent (Levelt, 1968; Rubin and Hupé, 2004; Pressnitzer and Hupé, 2005), and that the initial percept is the grouped one (Anstis and Saida, 1985; Shamma, 2008; Hupé and Pressnitzer, 2012). We formalize these into a theoretical framework that makes explicit the link between averaging over random switch times between states, and the dynamic description of the probability of being in one state, which is the buildup function.

Furthermore, our general, non-mechanistic, ARP model also provides a cautionary message with regard to mechanistic hypotheses for buildup: Buildup does not necessarily imply some sort of dynamical adaptation or accumulation of evidence across epochs. All switches after the first one follow rules that are fixed with time thereafter. There is no internal dynamical variable (such as one labeled as “adaptation” or “evidence”) that governs the timecourse of buildup. In other words, at the moment a switch into state *Z* = *i*, is made, the random dwell time in that state, before switching into state, *Z* = NOT *i*, is always drawn from the same bucket. From this viewpoint, the timecourse of the buildup function could be merely a reflection of the relaxation of the ensemble-average of the inherently stochastic state variable *Z*(*t*) to a steady-state value.

A heuristic analogy is provided by the fraction of open voltage-gated K^+^-channels in the Hodgkin and Huxley model for K^+^-conductance in neural membranes (Hodgkin and Huxley, 1952). When one observes individual K^+^-channel behavior, as in the on-cell patch clamp method of Neher and Sakmann (1976), one can see that the single-channel contribution to the population gating variable is made by a dichotomous random variable, just like our *Z* (*t*), which we will call *N*(*t*). The critical point is that rate constants (probability per unit time) underlying the transition from *N* = *i* to *N* = NOT *i* depend instantaneously on the membrane voltage, *V*(*t*). The consequences at the level of the ensemble average are revealed when one measures the net fraction of maximal K^+^-conductance, in which contributions made by thousands of K^+^-channels in the membrane of the neuron. A highly germane experiment is the classical voltage-clamp experiment (Hodgkin and Huxley, 1952); the fractional conductance is analogous to our buildup function. In a voltage-clamp experiment in which the membrane voltage is first held fixed at a level below the resting membrane voltage, and then stepped up instantaneously to a “command” voltage well above the resting voltage at time zero and then held fixed, the overall fractional conductance follows a sigmoidal time course, starting near zero, and reaching a plateau between zero and 1. The dynamics are completely determined by the command voltage at the instant of the voltage step. The timecourse reflects the relaxation of the overall conductance to a level governed by the command voltage. The important point is that each individual channel is undergoing independent state transitions (one state of which corresponds to an open channel) with probabilities per unit time that are fixed forever after the step. The timecourse of the conductances stems from the fact that the transition rates going toward the open-channel state are larger, and the transition rates leading to all closed-channel states are smaller, at the command potential established at *t* = 0 than they were for *t* < 0. It seems that the notions of adaptation, accumulation, and analogous processes are not relevant here. In other words, there is no external function of time driving the evolution of the channel ensemble.

## Concluding remarks

We developed the statistical ARP model to demonstrate that buildup does not necessarily implicate accumulation or adaptation as dynamic mechanisms. Rather, the buildup function may simply reflect the evolution of probability after trial-averaging for state 1 having started in state 0 under random, independently-timed, switching between states.

Additional data, say for non-stationary conditions or strong correlations between percept durations, may help to distinguish among mechanistic models, but buildup alone provides insufficient support. Even with a transiently perturbed stimulus, the subsequent relaxation of probability to a steady value can be described with the ARP model. The ARP model can account for a wide range of buildup time courses, monotonic or not. We illustrated that non-monotonic and damped cyclic buildup occurs in the ARP model when the duration distributions are sharply localized. The competition model in the noisy oscillator regime can have small variance durations. Given that nearly all reported buildup functions are monotonic [with the possible exception of those in Anstis and Saida (1985)], our results support the suggestion in Shpiro et al. (2009) that the dynamics of perceptual switching favor a qualitative basis of noise-driven attractor dynamics over noisy oscillator dynamics—but without speaking for a specific neuronal mechanism. Our ARP model provides a novel description and insight that links transient dynamics of the buildup function to any underlying steady state process that generates perceptual alternations, and it produces surprisingly good predictions with minimal assumptions.

### Conflict of interest statement

The authors declare that the research was conducted in the absence of any commercial or financial relationships that could be construed as a potential conflict of interest.

## References

[B1] Anstis S. Saida S. (1985). Adaptation to auditory streaming of frequency-modulated tones. J. Exp. Psychol. Hum. Percept. Perform. 11, 257–271 10.1037/0096-1523.11.3.257

[B2] Bee M. A. Micheyl C. Oxenham A. J. Klump G. M. (2010). Neural adaptation to tone sequences in the songbird forebrain: patterns, determinants, and relation to the build-up of auditory streaming. J. Comp. Physiol. A Neuroethol. Sens. Neural Behav. Physiol. 196, 543–557. 10.1007/s00359-010-0542-4 20563587 PMC2909344

[B3] Borsellino A. De Marco A. Allazetta A. Rinesi S. Bartolino B. (1972). Reversal time distribution in the perception of visual ambiguous stimuli. Kybernetik 10, 139–144. 10.1007/BF00290512 5021011

[B4] Bracewell R. (2000). The Fourier Transform and its Applications. New York, NY: McGraw-Hill Higher Education.

[B5] Bregman A. S. (1978). Auditory streaming is cumulative. J. Exp. Psychol. Hum. Percept. Perform. 4, 380–387. 10.1037/0096-1523.4.3.380 681887

[B6] Carlyon R. P. (2004). How the brain separates sounds. Trends Cogn. Sci. 8, 465–471. 10.1016/j.tics.2004.08.008 15450511

[B7] Carlyon R. P. Cusack R. Foxton J. M. Robertson I. H. (2001). Effects of attention and unilateral neglect on auditory stream segregation. J. Exp. Psychol. Hum. Percept. Perform. 27, 115–127. 10.1037/0096-1523.27.1.115 11248927

[B8] Cox D. R. (1962). Renewal Theory. New York, NY: John Wiley and Sons.

[B9] Cusack R. Deeks J. Aikman G. Carlyon R. P. (2004). Effects of location, frequency region, and time course of selective attention on auditory scene analysis. J. Exp. Psychol. Hum. Percept. Perform. 30, 643–656. 10.1037/0096-1523.30.4.643 15301615

[B10] Deike S. Heil P. Böckmann-Barthel M. Brechmann A. (2012). The build-up of auditory stream segregation: a different perspective. Front. Psychol. 3:461. 10.3389/fpsyg.2012.00461 23118731 PMC3484653

[B11] Denham S. L. Bendixen A. Mill R. Tóth D. Wennekers T. Coath M.. (2012). Characterising switching behaviour in perceptual multi-stability. J. Neurosci. Methods 210, 79–92. 10.1016/j.jneumeth.2012.04.004 22525854

[B12] Denham S. L. Gyimesi K. Stefanics G. Winkler I. (2010). Stability of perceptual organisation in auditory streaming, in The Neurophysiological Bases of Auditory Perception, 1st Edn., eds Lopez-Poveda E. A. Palmer A. R. Meddis R. (New York, NY: Springer Science+Business Media), 477–487.

[B13] Hodgkin A. Huxley A. (1952). A quantitative description of membrane current and its application to conduction and excitation in nerve. J. Physiol. 117, 500–544. 12991237 10.1113/jphysiol.1952.sp004764 PMC1392413

[B14] Huguet G. Rinzel J. Hupé J. (2014). Noise and adaptation in multistable perception: noise drives when to switch, adaptation determines percept choice. J. Vis. 14, 1–24. 10.1167/14.3.19 24627459

[B15] Hupé J.-M. Pressnitzer D. (2012). The initial phase of auditory and visual scene analysis. Philos. Trans. R. Soc. Lond. B Biol. Sci. 367, 942–953. 10.1098/rstb.2011.0368 22371616 PMC3282313

[B16] Hupé J.-M. Rubin N. (2003). The dynamics of bi-stable alternation in ambiguous motion displays: a fresh look at plaids. Vis. Res. 43, 531–548. 10.1016/S0042-6989(02)00593-X 12594999

[B17] Kakubava R. (2008). Analysis of alternating renewal process. R&RATA 1, 77–83.

[B18] Karlin S. (1958). The application of renewal theory to the study of inventory policies, in Studies in the Mathematical Theory of Inventory and Production, eds Arrow K. Karlin S. Scarf H. (Stanford, CA: Stanford University Press), 270–297.

[B19] Kondo H. M. Kashino M. (2009). Involvement of the thalamocortical loop in the spontaneous switching of percepts in auditory streaming. J. Neurosci. 29, 12695–12701. 10.1523/JNEUROSCI.1549-09.2009 19812344 PMC6665088

[B20] Laing C. R. Frewen T. Kevrekidis I. G. (2010). Reduced models for binocular rivalry. J. Comput. Neurosci. 28, 459–476. 10.1007/s10827-010-0227-6 20182782

[B21] Levelt W. (1968). On Binocular Rivalry. The Hague: Mouton.

[B22] Micheyl C. Tian B. Carlyon R. P. Rauschecker J. P. (2005). Perceptual organization of tone sequences in the auditory cortex of awake macaques. Neuron 48, 139–148. 10.1016/j.neuron.2005.08.039 16202714

[B23] Mill R. W. Bõhm T. M. Bendixen A. Winkler I. Denham S. L. (2013). Modelling the emergence and dynamics of perceptual organisation in auditory streaming. PLoS Comput. Biol. 9:e1002925. 10.1371/journal.pcbi.1002925 23516340 PMC3597549

[B24] Moreno-Bote R. Rinzel J. Rubin N. (2007). Noise-induced alternations in an attractor network model of perceptual bistability. J. Neurophysiol. 98, 1125–1139. 10.1152/jn.00116.2007 17615138 PMC2702529

[B25] Mortensen R. E. (1990). Alternating renewal process models for electric power system loads. Automat. Control IEEE Trans. 35, 1245–1249 10.1109/9.59801

[B26] Neher E. Sakmann B. (1976). Single-channel currents recorded from membrane of denervated frog muscle fibres. Nature 260, 799–802. 10.1038/260799a0 1083489

[B27] Pastukhov A. García-Rodríguez P. E. Haenicke J. Guillamon A. Deco G. Braun J. (2013). Multi-stable perception balances stability and sensitivity. Front. Comput. Neurosci. 7:17. 10.3389/fncom.2013.00017 23518509 PMC3602966

[B27a] Pham-Gia T. Turkkan N. (1999). System availability in a gamma alternating renewal processes. Nav. Res. Log. 46, 822–844.

[B28] Pressnitzer D. Hupé J. (2005). Is Auditory Streaming a Bistable Percept? Budapest: Forum Acusticum.

[B29] Pressnitzer D. Hupé J.-M. (2006). Temporal dynamics of auditory and visual bistability reveal common principles of perceptual organization. Curr. Biol. 16, 1351–1357. 10.1016/j.cub.2006.05.054 16824924

[B30] Pressnitzer D. Sayles M. Micheyl C. Winter I. M. (2008). Perceptual organization of sound begins in the auditory periphery. Curr. Biol. 18, 1124–1128. 10.1016/j.cub.2008.06.053 18656355 PMC2559912

[B31] Pressnitzer D. Suied C. Shamma S. (2011). Auditory scene analysis: the sweet music of ambiguity. Front. Hum. Neurosci. 5:158. 10.3389/fnhum.2011.00158 22174701 PMC3237025

[B32] Rubin N. Hupé J.-M. (2004). Dynamics of perceptual bi-stability: plaids and binocular rivalry compared, in Binocular Rivalry, eds Alais D. Blake R. (Cambridge, MA: MIT Press), 1–13.

[B33] Shamma S. (2008). On the emergence and awareness of auditory objects. PLoS Biol. 6:e155. 10.1371/journal.pbio.0060155 18578570 PMC2435155

[B34] Shpiro A. Moreno-bote R. Rubin N. Rinzel J. (2009). Balance between noise and adaptation in competition models of perceptual bistability. J. Comput. Neurosci. 27, 37–54. 10.1007/s10827-008-0125-3 19125318 PMC2913428

[B35] Stinchcombe A. Peskin C. Tranchina D. (2012). Population density approach for discrete mRNA distributions in generalized switching models for stochastic gene expression. Phys. Rev. E 85, 1–12. 10.1103/PhysRevE.85.061919 23005139

[B36] Van Noorden L. P. A. S. (1975). Temporal Coherence in the Perception of Tone Sequences. Doctoral dissertation, Technical University Eindhoven, Eindhoven.

[B37] Von Grünau M. Dubé S. (1993). Ambiguous plaids: switching between coherence and transparency. Spat. Vis. 7, 199–211. 10.1163/156856893X00360 8251434

[B38] Wilbur W. Rinzel J. (1982). An analysis of Stein's model for stochastic neuronal excitation. Biol. Cybern. 45, 107–114 10.1007/BF00335237 7138956

[B39] Wilson H. R. (2003). Computational evidence for a rivalry hierarchy in vision. Proc. Natl. Acad. Sci. U.S.A. 100, 14499–14503. 10.1073/pnas.2333622100 14612564 PMC283620

[B40] Wilson H. R. Cowan J. D. (1972). Excitatory and inhibitory interactions in localized populations of model neurons. Biophys. J. 12, 1–24. 10.1016/S0006-3495(72)86068-5 4332108 PMC1484078

